# Influenza Virus Induces Inflammatory Response in Mouse Primary Cortical Neurons with Limited Viral Replication

**DOI:** 10.1155/2016/8076989

**Published:** 2016-07-21

**Authors:** Gefei Wang, Rui Li, Zhiwu Jiang, Liming Gu, Yanxia Chen, Jianping Dai, Kangsheng Li

**Affiliations:** Department of Microbiology and Immunology, Guangdong Provincial Key Laboratory of Infectious Disease and Molecular Immunopathology, Shantou University Medical College, Shantou 515041, China

## Abstract

Unlike stereotypical neurotropic viruses, influenza A viruses have been detected in the brain tissues of human and animal models. To investigate the interaction between neurons and influenza A viruses, mouse cortical neurons were isolated, infected with human H1N1 influenza virus, and then examined for the production of various inflammatory molecules involved in immune response. We found that replication of the influenza virus in neurons was limited, although early viral transcription was not affected. Virus-induced neuron viability decreased at 6 h postinfection (p.i.) but increased at 24 h p.i. depending upon the viral strain. Virus-induced apoptosis and cytopathy in primary cortical neurons were not apparent at 24 h p.i. The mRNA levels of inflammatory cytokines, chemokines, and type I interferons were upregulated at 6 h and 24 h p.i. These results indicate that the influenza virus induces inflammatory response in mouse primary cortical neurons with limited viral replication. The cytokines released in viral infection-induced neuroinflammation might play critical roles in influenza encephalopathy, rather than in viral replication-induced cytopathy.

## 1. Background

Influenza virus is an enveloped, multiple-segmented, negative-stranded RNA virus that mainly infects the respiratory tract and causes health problems ranging from common cold-like symptoms to severe infections such as pneumonia. Influenza virus is also associated with many neurological complications such as encephalopathy/encephalitis syndrome, Reye's syndrome, hemorrhagic shock, encephalopathy syndrome, and acute necrotizing encephalopathy [1–3].

The microenvironment of central nervous system (CNS) is highly specialized and is considered an immune-privileged site due to the CNS-driven passive interactions with the immune system [4, 5]. These mechanisms involve neuron and glial cells including microglia, astrocytes, and oligodendrocytes [5]. Microglia belong to resident phagocytic cells which function as the first line of CNS defense, and astrocytes are the principal source of cytokines secretion upon stress, injury, and infection. Microgliosis and astrocytosis play roles in a spectrum of neurodegenerative disorders [6, 7]. Neurons have traditionally been implicated as the sole targets of microglia cytotoxicity and innocent victims of overactivated immune cells. Recent researches, however, have demonstrated that neurons might host and regulate innate and adaptive immune responses to counter viral infection in the CNS [8–10].

Accumulating evidences have shown that the RNA and antigen of the neurovirulent influenza virus can be detected in the neurons of human, mice, and birds [11–13]. Influenza virus enters CNS and induces neuroinflammation and neurodegeneration [7]. Previous studies demonstrated that both human H1N1 and avian H5N1 influenza viruses infect microglia, astrocytes, and neuronal cell lines* in vitro*, inducing production of proinflammatory cytokines and ultimately leading to cell apoptosis or cytopathy [14–16].

The present study was to determine the potential role of neurons in the innate immune response to the influenza virus. We examined cytokine expression, viral susceptibility and production, cell viability, and apoptosis in the infected neurons. We found that neurons upregulated the expression of cytokines, chemokines, and type I interferons (IFNs) to counter influenza infection. In addition, the nuclear factor kappa-light-chain-enhancer of activated B cells (NF-*κ*B) signaling was not activated in neurons after influenza virus infection.

## 2. Materials and Methods

### 2.1. Animals

This study was preapproved by the Ethical Committee of Shantou University Medical College. Specific pathogen-free C57 BL/6 18-day-old pregnant mice were purchased from Shantou University Medical College Laboratory Animal Center (Shantou, Guangdong, China).

### 2.2. Cell Culture

C57 BL/6 female mice were mated with male mice, and vaginal plugs were checked every morning. Embryonic day (E0.5) refers to the day that a vaginal plug was found. To collect embryos, the pregnant females were euthanized and E17.5 embryos were dissected from the uteri. Cortical neurons from the embryos were isolated as previously described [17]. Briefly, cortices were dissociated in phosphate-buffered saline (PBS, 0.01 M, pH 7.4) and plated (1 × 10^6^ cells/mL) on poly-D-lysine- (PDL-) coated plates. The neurons were grown in plating medium containing Dulbecco's Modified Eagle's Medium with 10% fetal bovine serum and antibiotics (100 units/mL penicillin, 100 *μ*g/mL streptomycin, and 0.25 *μ*g/mL amphotericin B; Gibco BRL). Plating medium was changed into neurobasal medium with 2% B27 supplemented and 0.5 mM Glutamax and antibiotics after 6 h. About one-half of the culture medium was replaced every 3-4 days. The cultures were maintained at 37°C in a 5% CO_2_ humidified atmosphere.

### 2.3. Viruses and Viral Titers

Two influenza virus H1N1 strains, A/PR/8/34 (PR8) and A/Shantou/169/2006 (ST169), were used in this study. The titers of PR8 and ST169 were diluted into 2 × 10^6^ PFU (plaque forming units)/mL. Viral titers were determined by plaque assay on Madin-Darby canine kidney (MDCK) as previously described [14]. Briefly, 90% of confluent MDCK cell monolayers were infected with 10-fold dilutions of influenza virus in a total volume of 1 mL Minimum Essential Medium (MEM)/0.2% BSA for 1 h in a 6-well plate. After washing, cells were covered with an overlay of MEM cell culture medium containing 0.9% low melting point agarose. Cells were incubated at 37°C under 5% CO_2_ and plaque formation was analyzed 3 days postinfection (p.i.).

### 2.4. Cell Infections

The neurons were washed twice with PBS and infected with viruses at a multiplicity of infection (MOI) of 2. One hour postinfection, cells were washed once with PBS and cultured with fresh neuron culture medium. Untreated control cells were included in each independent experiment as negative controls. The cell culture supernatants of infected neurons were collected at 0, 6, and 24 h p.i., and their cell suspensions were collected at 0, 6, and 24 h p.i. and stored at −80°C until subsequent use.

### 2.5. Real-Time PCR

Total RNA was extracted from virus infected and control samples using Trizol reagent (Invitrogen, USA) according to the manufacturer's instructions. First-strand cDNA synthesis was performed with the M-MLV enzyme (Invitrogen, USA) in a final volume of 20 *μ*L. The samples were diluted with DEPC (diethylpyrocarbonate) water to a 1 : 25-fold concentration. Real-time PCR was performed using the 7300 Fast Real-Time PCR system (ABI, USA). Quantification of the target genes was performed with 2x platinum quantitative PCR supermix-UDG kit (Invitrogen, USA) and specific primer sets (Qiagen, Germany) according to the manufacturer's instructions. The specificity of the SYBR Green PCR signal was confirmed by melting curve analysis. In each experiment, mouse *β*-actin mRNA was amplified as a control. Each threshold cycle (Ct) value was calculated by taking an average of the values obtained from triplicate samples. To examine virus infection and neuronal viral growth kinetics, viral mRNA, viral RNA (vRNA), and complementary (cRNA) levels in infected neurons were detected by real-time PCR at 6 h and 24 h p.i., respectively.

### 2.6. Enzyme Linked Immunosorbent Assay of IL-6 and TNF-*α*

Production of interleukin-6 (IL-6) and tumor necrosis factor-alpha (TNF-*α*) in the supernatants of mouse primary neurons was determined using specific enzyme linked immunosorbent assay (ELISA) kits (Dakewe, China) according to the manufacturer's instructions. The culture supernatants of the infected and uninfected control cells were irradiated with UVP CX-2000 Crosslinker (UVP, Upland, CA, USA) for 15 min to make virus ineffective before subjecting to ELISA. No infective virus particles were detected by plaque assay after ultraviolet (UV) irradiation. The dose of UV light used did not affect cytokine concentration, as confirmed by our previous experiments.

### 2.7. Cell Viability and Apoptosis Assay

Cell viability was assessed with a cell counting kit-8 (Dojindo Laboratories, Kumamoto, Japan). Cells were seeded and grown for 5 days in 96-well plates at a density of 1 × 10^5^ cells per well in neural culture medium prior to any treatment. After treating with influenza A virus (MOI = 2), 10 *μ*L of kit reagent was added and the solution was incubated for another 2 h. Cell viability was determined by scanning with a microplate reader at 450 nm. The results were expressed relative to the control values specified in each experiment and were subjected to statistical analysis. A quantitative enzymatic activity assay was performed according to the instructions of the caspase-3 activity assay kit (Roche Applied Science, Mannheim, Germany). Absorbance was measured at 505 nm.

### 2.8. Immunofluorescence Staining

For immunofluorescence staining, cells were fixed in 4% paraformaldehyde and penetrated with 0.2% Triton X-100 and 0.04% SDS in PBS. The cells were incubated with 1 : 500 diluted rabbit anti-mouse beta-tubulin III antibody (Santa Cruz, CA, USA) for neuron identification or 1 : 1000 diluted rabbit anti-mouse NF-*κ*B p65 antibody (Cell Signaling, Danvers, MA, USA) for NF-*κ*B location. Later, the cells were incubated with fluorescence-labeled secondary antibody (Beyotime, Haimen, China) and 1 *μ*g/mL Hoechst 33258. The processed cells were imaged by fluorescence microscopy.

### 2.9. Hemagglutination Assay

The culture supernatants of infected neurons were gradient diluted 1 : 2 in PBS (0.01 M, pH 7.4) in a serial 2-fold dilution. The diluted supernatants (50 *μ*L/well) were mixed with the equal volumes of 1% guinea pig red blood cells in V-shaped 96-well microtiter plates to determine virus hemagglutinin (HA) titers.

### 2.10. Statistical Analysis

Student's *t*-test was used to compare the difference between virus infected and uninfected cells. The data were presented as mean ± SD. The differences were considered statistically significant at *P* < 0.05. The statistical analysis was done using the SPSS 13.0 for Windows (Chicago, IL, USA).

## 3. Results

### 3.1. Infection of Primary Neurons by Human Influenza Viruses

**Figure 1:**
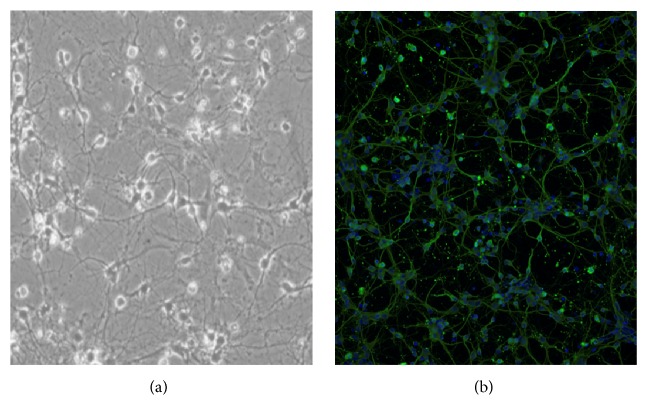
Neurons stained with beta-tubulin III (green); purity is >95% (200x magnification).

The cultured cells showed a typical neuronal morphology (Figure 1(a)). Hoechst 33258- and *β*-tubulin III immunostain displayed that the purity of primary mouse cortical neurons was over 95% (Figure 1(b)).

**Figure 2:**
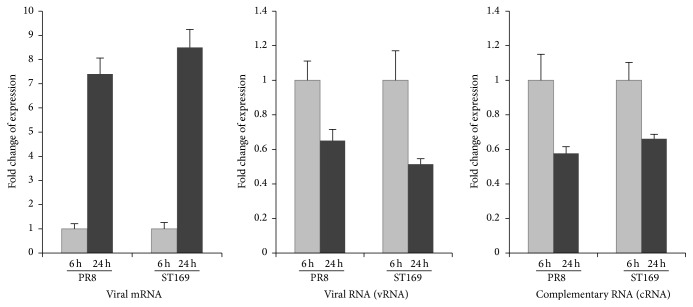
Influenza A viral matrix mRNA, vRNA, and cRNA were measured by real-time PCR. The relative amounts were represented as the ratio of mRNA, vRNA, and cRNA expression in the infected neurons at 24 h p.i. versus at 6 h p.i.

Primary mouse neurons were infected with two strains of H1N1 (PR8 and A/Shantou/169/06) viruses. The expression of viral matrix mRNA was detectable in the neurons at 6 h p.i. and increased at 24 h p.i. In contrast, the expression of vRNA and cRNA in neurons was decreased postinfection (Figure 2). HA titers of the supernatants from infected neurons at 6 and 24 h p.i. were determined. Undetected HA titer confirmed that influenza virus progeny was absent or very low in the supernatants.

**Figure 3:**
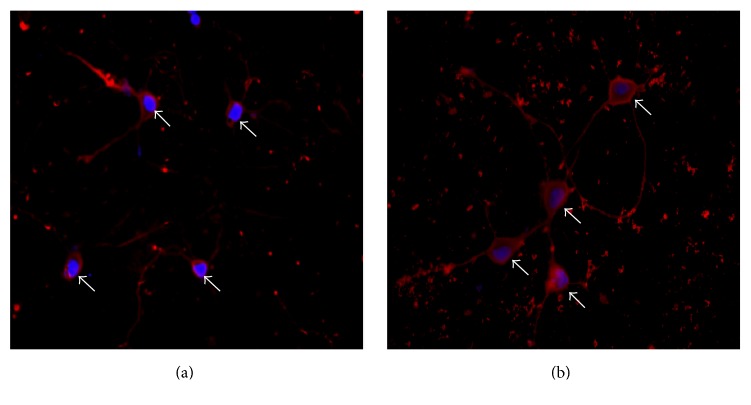
The determination of NF-*κ*B location using NF-*κ*B p65 immunostaining. NF-*κ*B p65 (red) was mainly retained in the cytosol, in both uninfected (a) and infected (b) neurons, suggesting NF-*κ*B pathway was not activated by virus infection. Nuclei were stained with Hoechst 33258 (blue) (400x magnification).

Immunostaining of NF-*κ*B showed that NF-*κ*B located in cytosol in uninfected neurons and did not translocate into nuclei after virus infection (Figure 3).

### 3.2. Induction of Cytokines following Infection

**Figure 4:**
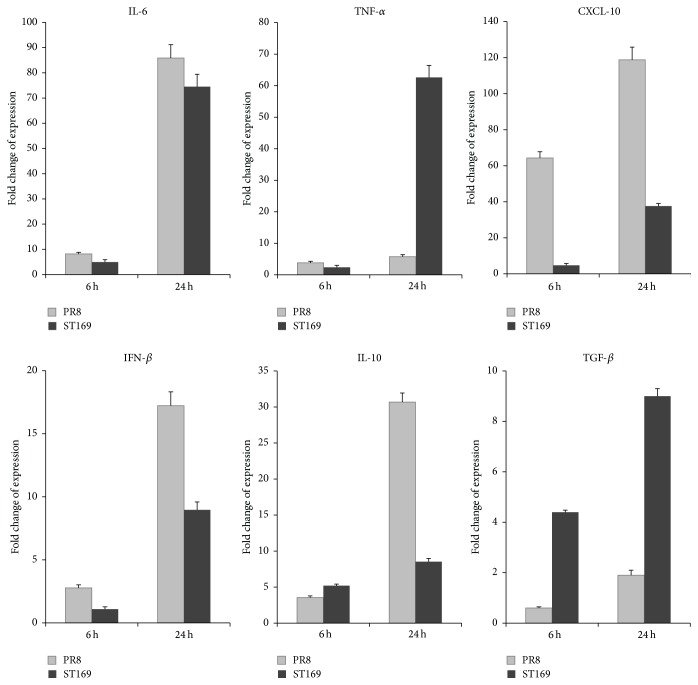
Induction of cytokines in primary mouse cortical neurons after influenza A virus infection at an MOI of 2. Mock-infected neurons served as controls. The expression levels of target gene were determined by RT-PCR, normalized to housekeeping gene *β*-actin, and represented as fold change difference of mRNA levels relative to uninflected neurons.

To analyze the response of cytokines in infected neurons, the gene expression of proinflammatory cytokines, chemokines, antivirus cytokines, and anti-inflammatory cytokines was analyzed by real-time PCR at 6 and 24 h p.i. The results showed that the expression of IL-6, TNF-*α*, CXCL-10, IFN-*β*, IL-10, and TGF-*β* was increased significantly after PR8 and ST169 infection (Figure 4). Interestingly, ST169 upregulated proinflammatory cytokine TNF-*α* expression more sharply than PR8. Moreover, the level of anti-inflammatory cytokine expression (IL-10 and TGF-*β*) increased dramatically after PR8 infection but increased moderately after ST169 infection. It might suggest that the severity of neuroinflammation depends upon the viral strain.

### 3.3. Viability of Neurons after Influenza Virus Infection

**Figure 5:**
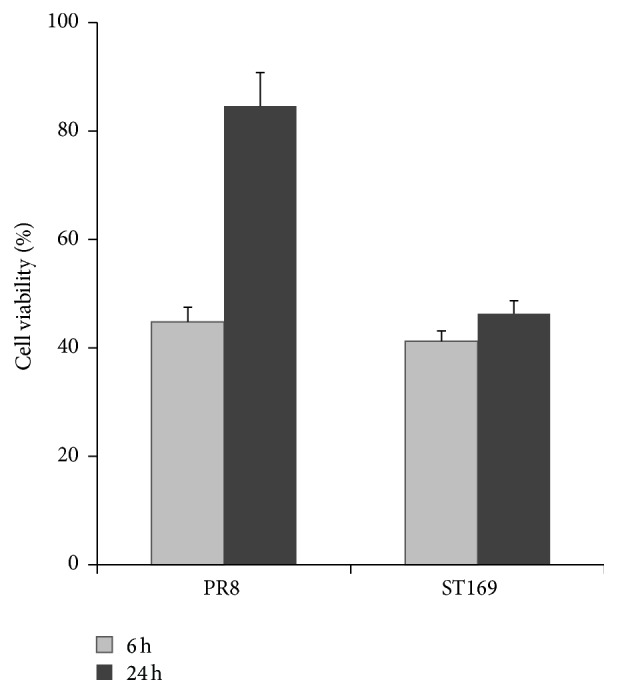
Changes in viability in primary cortical neurons after infection with influenza A virus. Neurons were infected with H1N1 (PR8 or ST169) at an MOI of 2. Mock-infected neurons were the controls. Cell viability was assessed with a cell counting kit-8. Results were represented from three separate experiments.

To determine the effects of influenza virus on neuron viability, cell counting kit-8 assay was conducted. As shown in Figure 5, the viability of neurons dropped to 44.5% and 41% at 6 h p.i. upon infection of PR8 and ST169, respectively. Neuronal cell recovery could be detected in PR8 inflected neurons at 24 h p.i. (cell viability rose from 44.5% to 84.1%), whereas moderate recovery of cell viability could be observed in ST169 inflected neurons.

Viral infection-induced cytopathic effects were not observed. Consistently, we also found the level of caspase-3 in infected neurons did not alter significantly, comparing to the uninfected controls (data not shown). Both cytopathy and apoptosis of neuron were not induced directly by influenza virus infection.

## 4. Discussion

Some variant strains of human influenza virus (WSN33 and pH1N1) and avian influenza (H5 and H7 subtypes) have been shown to possess neurotropic tendency [18]. The potential routes for influenza A virus spreading into CNS are hematogenous spread or neural spread, which is related to the subtype of influenza A virus [19, 20]. Although influenza RNA was not always detected, neurovirulent influenza viruses are still considered to play an important role in neurodegenerative disease or encephalitis [7, 21, 22].

Our present work also showed that influenza virus (low pathogenic H1N1) infected cortical neurons* in vitro* without significant increase of new progeny virus, which was consistent with previous studies [15]. However, by examining influenza A virus genomic RNA and cRNA expression levels, we found that their levels did not significantly change at 6 h to 24 h p.i., indicating that viral genomic RNA replication was efficiently inhibited in the neuron. It is well known that NF-*κ*B signaling pathway is required for efficient influenza A virus replication and the differential regulation of influenza virus RNA synthesis [23]. Therefore, absence of NF-*κ*B activation in our work might contribute to the lack of influenza virus replication. In addition, innate immune pathways are the first defense response for the immediate control and eventual clearance of pathogens, which may also play a role in efficiently inhibiting influenza virus replication.

Apoptosis is an important defense mechanism against intracellular pathogen infection (especially viral infection) through curb pathogen replication and dissemination. Virus-induced neuronal apoptosis has been demonstrated* in vitro* and* in vivo* by animal experiments previously [24]. However, in the present work, virus-induced apoptosis was not apparent, suggesting that influenza virus causes neuron injury by indirect immunopathogenesis instead of direct viral damage.

Cytokines play a dual role in CNS virus infection. Whereas moderate innate mediators mediate a protected response which leads to viral clearance and tissue recovery, however, uncontrolled production of proinflammatory cytokines could result in immunopathogenesis [25]. It is well known that influenza virus infection can induce a cascade of cytokines including proinflammatory and anti-inflammatory cytokines, chemokines, and antivirus cytokines [26]. Our previous study also showed that the proinflammatory cytokines IL-6 and TNF-*α* were upregulated in microglia and astrocytes at the mRNA and protein levels in the early phase (6 h) as well as in the late phase (24 h) after H1N1 infection* in vitro* [13]. The present study showed that IL-6 and TNF-*α* were also upregulated at the mRNA level after H1N1 exposure, in accordance with previous studies. Chemokine CXCL10, as a signaling mediator, can activate microglia and direct them to the lesions and is constitutively expressed by neurons [27]. Our results showed that CXCL10 mRNA expression levels were significantly upregulated at 6 h and 24 h p.i., suggesting that it might contribute to the neuropathogenesis after viral infection. INFs are hallmarks of the antivirus response and are critical in the host defense to viruses by modulating the innate and adaptive immune response. Our study showed that IFN-*β* mRNA levels were upregulated at 24 h p.i., suggesting neurons' antivirus effect was activated, since neurons could take an active part in the anti-influenza defense by being both IFN-*β* producers and responders [28]. As the important mediators of negative regulation, anti-inflammatory cytokines such as IL-10 and TGF-*β* play important roles in maintaining the balance between protective immunity and the development of immune pathology in the context of infectious disease [29]. IL-10 and TGF-*β* mRNA levels increased at 24 h p.i. because neuraminidase of most influenza A viruses can convert latent TGF-*β* to active TGF-*β*, which plays a pivotal role in protecting the host from influenza pathogenesis [30]. Our results demonstrated that neurons might protect themselves by upregulating IL-10 and TGF-*β*.

Generally, the interactions between virus strains and cells are complex and the specific antivirus immune response is both virus- and cell-dependent. Influenza virus enters CNS and infects and activates the glial cells, which may subsequently induce neuron injury by causing neuroinflammation and neurodegeneration. In this study, we demonstrate that influenza virus replication was limited in purified cultures of neurons and direct virus-induced neuron apoptosis was not apparent. However, the cytokines were upregulated by viral infection, which might induce microgliosis and astrocytosis. The cytokines released in viral infection-induced neuroinflammation may play key roles in influenza encephalopathy, rather than in viral replication-induced cytopathy.
